# Feasibility of knee magnetic resonance imaging protocol using artificial intelligence-assisted iterative algorithm protocols: comparison with standard MRI protocols

**DOI:** 10.3389/fmed.2024.1480196

**Published:** 2024-10-23

**Authors:** Hailong Liu, Yanxia Chen, Meng Zhang, Han Bu, Fenghuan Lin, Jun Chen, Mengqiang Xiao, Jie Chen

**Affiliations:** ^1^Department of Radiology, Zhuhai Hospital, Guangdong Provincial Hospital of Chinese Medicine, Zhuhai, China; ^2^Department of Radiology, Qujing Second People’s Hospital, Qujing, China

**Keywords:** iterative algorithm, knee, artificial intelligence, magnetic resonance imaging, acceleration technique

## Abstract

**Objective:**

To evaluate the image quality and diagnostic performance of AI-assisted iterative algorithm protocols (AIIA) in accelerated fast spin-echo magnetic resonance imaging (MRI) versus standard (SD) fast spin-echo MRI for clinical 3.0 T rapid knee scans.

**Materials and methods:**

The accelerated sequence, which includes fat-suppression proton density-weighted imaging (FS-PDWI), T2-weighted imaging (T2WI), and T1-weighted imaging (T1WI), was used in conjunction with the SD sequence in 61 patients who underwent MRI scans. SD images were processed using standard reconstruction techniques, while accelerated images utilized AIIA reconstruction. Quantitative assessments of image quality were conducted, measuring noise levels, signal-to-noise ratio (SNR) and contrast signal-to-noise ratio (CNR). Additionally, subjective evaluations were performed using a Likert five-point scale to assess image quality.

**Results:**

The SD group completed the entire knee scan in 466 s, while the AIIA group completed the scan in 312 s. Compared to the SD group, the AIIA group had a noticeably higher SNR of T1WI in the femur and subpatellar fat pad (*p* = 0.04, 0.001). On the other hand, T2WI femur SNR was noticeably higher in the SD group (*p* = 0.004). Measurements of SNR, CNR and other noise levels showed no statistically significant changes. Compared to the SD group, the AIIA group had significantly higher subjective image quality scores for every sequence (*p* < 0.05). There was a modest to large intraclass correlation value (ICC = 0.65–0.90) for the anomalies that were examined among readers. Both the AIIA and SD procedures were shown to have comparable diagnostic performance for meniscal and cruciate ligament rupture (*p* > 0.05).

**Conclusion:**

Images processed using AIIA reconstruction were acquired faster while maintaining comparable image quality and diagnostic capability, meeting the requirements for clinical diagnosis.

## 1 Introduction

Between 1990 and 2015, global life expectancy rose from 63.5 to 71.8 years (1, 2). Accompanying this demographic shift, the prevalence of knee disorders, particularly osteoarthritis—a leading cause of disability, has increased, significantly impacting individuals and society. These days, one of the most popular diagnostic procedures is MRI of the knee, which is incredibly useful for identifying structural problems (3). The long scan times of MRI limit patient throughput and the kinds of problems that can be solved by the technology. Extended scanning times can increase the likelihood of motion artifacts, particularly in patients experiencing knee pain, potentially leading to scan failures. Therefore, the development of advanced accelerated scanning protocols is a critical area for clinical research (4, 5).

The expenses of repeated MRI scans caused by motion artifacts are substantial, amounting to over $1.4 billion per year in the United States alone. Re-scans are required in 19.8% of instances and cost an additional $115,000. A primary cause is the discomfort or pain patients endure during lengthy scans, highlighting the urgency of reducing MRI scan times (6).

Iterative algorithms, traditionally utilized in CT scans, apply techniques in the projection space that adaptively use and modify anisotropic filters to balance noise suppression with detail preservation, performing spatial filtering multiple times to achieve the desired image quality (7, 8). Recently, these algorithms have been adapted for MRI to enhance image quality (9).

Advances in computer technology have facilitated the widespread clinical integration of artificial intelligence (AI) for disease diagnosis (10–12). Despite these acceleration efforts, there remains a potential for further reduction in scan times to manage the increasing patient volume. As AI technology continues to evolve, its role in imaging is becoming more prominent (13, 14).

**Figure 1:**
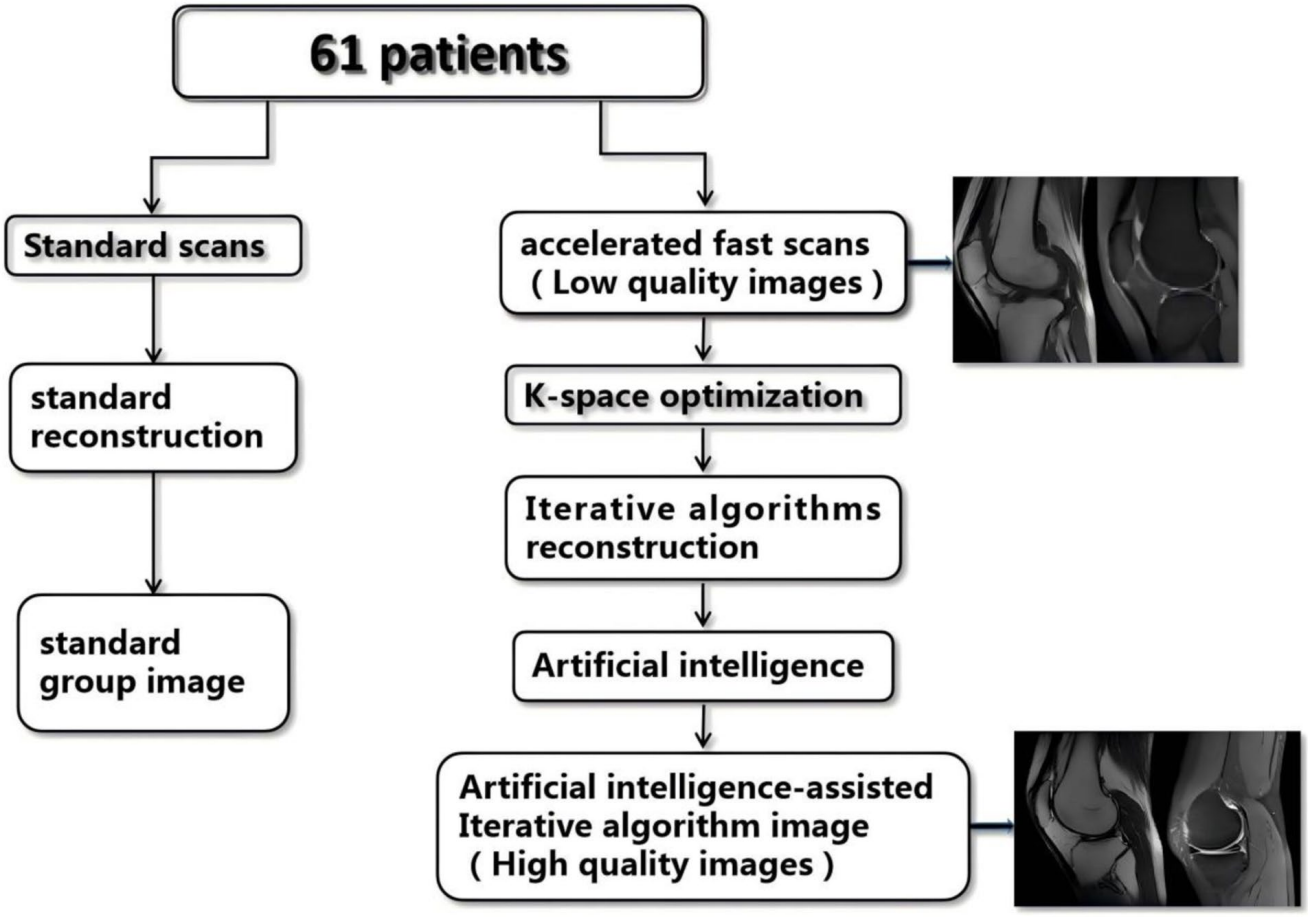
Flow chart of the study. SD, standard imaging; AIIA, artificial intelligence-assisted iterative algorithm protocols.

Artificial intelligence-assisted iterative algorithm protocols (AIIA) are well-established and effective for the nervous system (15). To increase SNR and iteratively reconstruct volumetric MR images, AIIA reconstruction makes use of statistical priors of noise distribution. This method improves the clarity and detail of MRI images captured using less-than-ideal scanning settings, like those with low resolution or speed (Figure 1). 3D patches are subdivided from the input dataset, which consists of magnetic resonance pictures. Each patch is assigned a unique similarity measure before being grouped into several features in a feature space for analysis. The assessment and differentiation of signal and noise are accomplished by merging information about patch similarity with estimations of noise statistics. This procedure is carried out again and again until the convergence requirements are satisfied.

Modulating picture sharpness or softness and improving edges are two examples of image-influencing factors that can be used to fine-tune the performance of basic iterative reconstruction techniques. In order to acquire the best processing settings for each image, an AI module compares the input photos to reference images. This guarantees that the output is very similar to the reference. To maximize the quality of the final image, these parameters are used in the iterative reconstruction procedure. The reference images are top-notch scans captured using the identical scanner that was utilized for the AIIA implementation.

To our knowledge, this is one of the first studies to assess the use of AIIA protocols in MRI of the knee joint. This study evaluates an AIIA acceleration technique in clinical knee imaging, aiming to reduce noise and enhance image sharpness. It compares the diagnostic performance of standard timing and standard reconstructed MRI images with accelerated timing and AIIA-reconstructed MRI images, exploring the potential application of AIIA technology in knee MRI scanning.

## 2 Materials and methods

### 2.1 Participants

The ZE2024-291-01 study was approved by the institutional research ethics committee of Guangdong Provincial Hospital of Chinese Medicine hospital. Every single participant gave their written informed consent. The xxx hospital enrolled 61 patients between the months of February 2024 and June 2024, comprising 22 males and 39 females. The age range was 18–84 years, with a mean of 50.39 ± 16.90 years. Patients who were 18 years old or older were eligible to participate, whereas those who were under the age of 18 or pregnant were not.

### 2.2 Scan protocols

A Signa HDx Echospeed, manufactured by General Electric Healthcare in the United States, a 3.0 T MRI machine, was used for the imaging. Clinically conventional knee MRI protocols comprised three-plane (2D-fat-suppression proton density-weighted imaging, or FS-PDWI) fast spin echo (FSE) sequences, T1WI, or T2WI, or sagittal FSE. Imaging parameters are detailed in Table 1. AIIA reconstruction (Medic Vision, IQMR) and SD reconstruction were performed for all knee MRI sequences (Figure 1).

**Table 1:** MRI sequences and parameters.

Sequence parameter	FOV (mm)	TR/TE (ms)	Matrix	ETL	Bandwidth (Hz)	NEX	Number slices	Accelerating factors	Fat suppression
T1WI	180*180	500/10	320*224	2	50	2	20	3	−
T2WI	180*180	4700/72	320*192	17	50	2	20	3	−
FS-PDWI	180*180	2800/38	320*224	10	50	4	20	3	+
T1WI (AIIA)	180*180	500/7	320*192	2	50	1	20	3	−
T2WI(AIIA)	180*180	4700/72	320*192	17	50	1	20	3	−
FS-PDWI(AIIA)	180*180	2800/38	320*224	10	50	2	20	3	+

AIIA, artificial intelligence-assisted iterative algorithm protocols; T1WI, T1-weighted image; T2WI, T2-weighted image; FS-PDWI, fat-suppression proton density weighted imaging; FOV, field of view; TR/TE, repetition time/echo time (ms); ETL, echo train length; NEX, number of excitation.

### 2.3 Image evaluation

#### 2.3.1 Objective indicators

**Figure 2:**
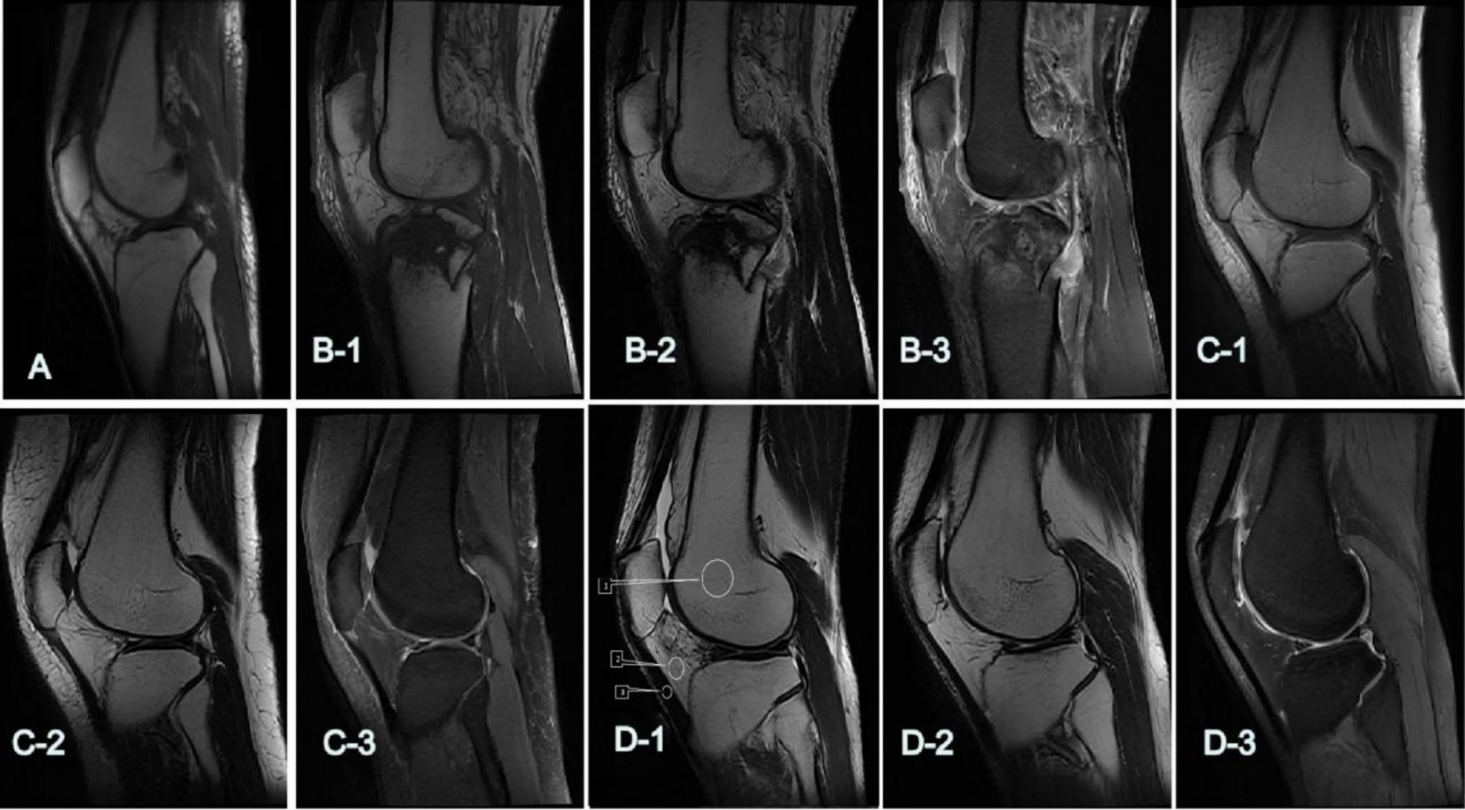
The 5-point Likert-type scale was utilized to assess the subjective quality of images. (A,B-1–D-1) Show the overall scores of T1WI: (A) 2: poor; (B-1) 3: qualified; (C-1) 4: good; (D-1) 5: excellent. (B-2–D-2) Show the overall scores of T2WI: (B-2) 3: qualified; (C-2) 4: good; (D-2) 5: excellent. (B-3–D-3) show the overall scores of FS-PDWI: (B-3) 3: qualified; (C-3) 4: good; (D-3) 5: excellent. D-1: T1WI, (ROIs) for the femoral bone marrow (BM, 1), infrapatellar fat pad (IPFP, 2), and patellar ligament (PL, 3).

To exclude areas with aberrant signal lesions, quantitative SNR evaluations were performed manually on a single slice using two circular regions of interest (ROIs) for the femoral bone marrow (BM), patellar ligament (PL), and infrapatellar fat pad (IPFP) at similar levels. The placement and measurement procedures for each sequence are shown in Figure 2D-1. Noise was calculated as the SD of the CT values for BM and IPFP. The signal-to-noise ratio (SNR) and contrast signal-to-noise ratio (CNR) were calculated as follows: SNR = average BM and IPFP CT value/image noise; CNR = (average IPFP attenuation − average PL attenuation)/image noise (16).

#### 2.3.2 Subjective indicators

**Figure 3:**
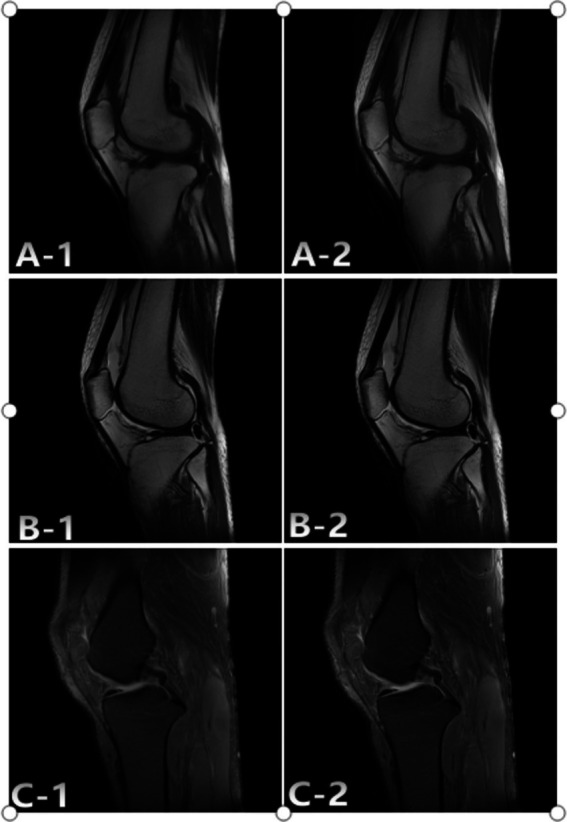
Representative case study 1. A 36-year-old woman with comminuted fracture of SD and AIIA scans. The SD: A-1; T1WI, B-1; T2WI, C-1; FS-PDWI. The overall score of was 3 points. The AIIA: A-2; T1WI, B-2; T2WI, C-2; FS-PDWI. The overall score of was 4 points. C-1 Ligament score 4 points, C-2 ligament score 5 points.

**Figure 4:**
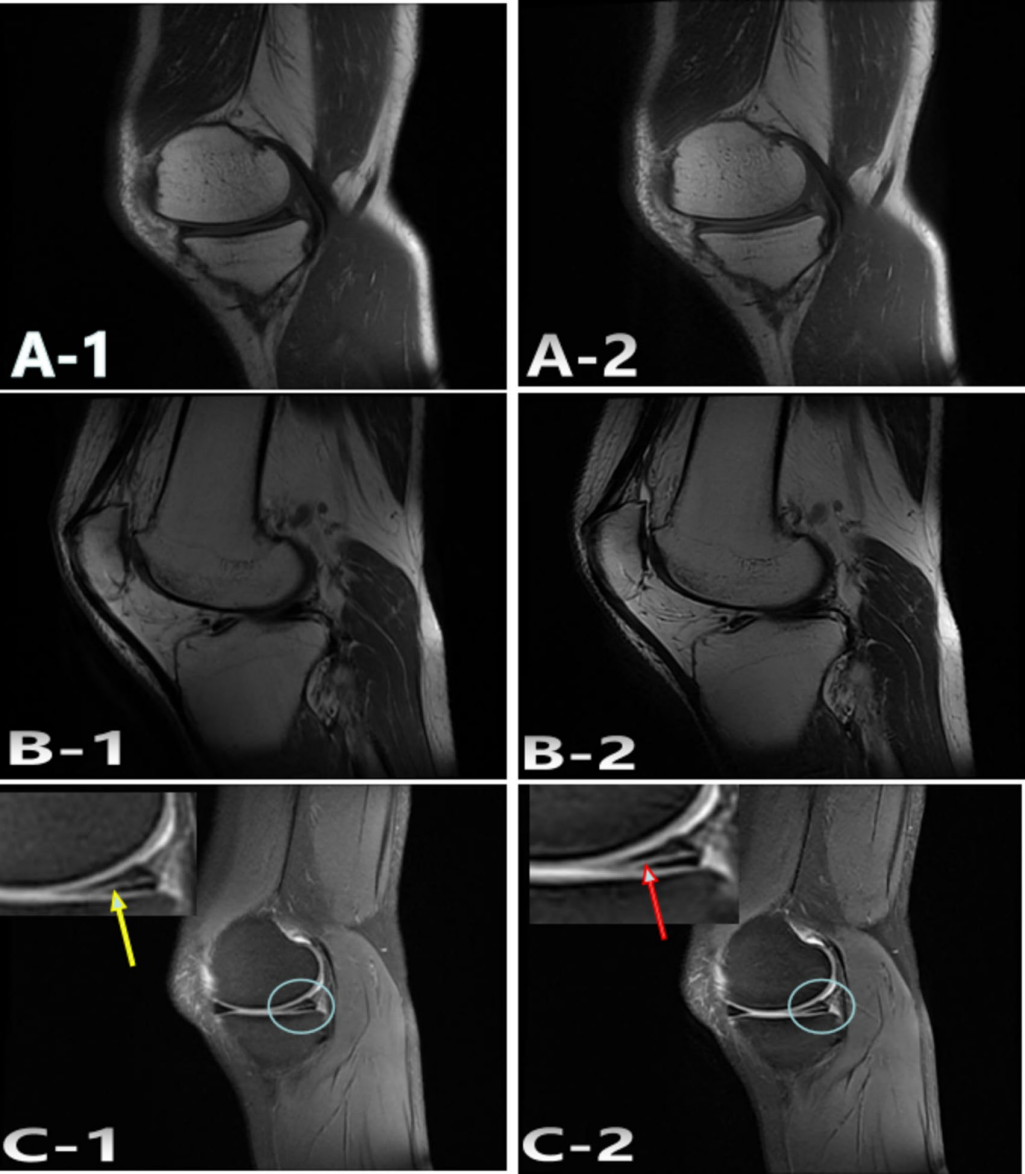
Representative case 2. A 47-year-old woman with undisplaced patella fracture underwent SD and AIIA scans. The SD: A-1; T1WI, B-1; T2WI, C-1; FS-PDWI. The overall score of was 4 points. The AIIA: A-2; T1WI, B-2; T2WI, C-2; FS-PDWI. The overall score of was 5 points. C-1 meniscus score 4 points, Torn posterior corner of medial meniscus (yellow arrow); C-2 meniscus score 5 points, Torn posterior corner of medial meniscus (red arrow).

A system called PACS (Guangzhou, China: Yi Lian Zhongrui Tu Information Technology Co., Ltd., Version 3.6) was used to evaluate the quality of the images (16, 17). Two highly experienced radiologists (HL and MX, with 22 and 20 years of expertise in musculoskeletal diagnosis, respectively) assessed the lesion detection, margin sharpness, artifacts, and overall picture quality using a 5-point scale. They did this separately, without access to clinical data. Figures 2–4 show the results of the image quality scoring: 1–5: terrible, fair, moderate, good, and perfect. The meniscus and ligaments (patellar, anterior, and posterior cruciate, collateral ligaments) were scored from 1 (poor, almost invisible) to 5 (excellent, very clear details). Since the FS-PDWI sequence is superior in assessing ligament and meniscus injuries compared to the T1WI and T2WI sequences (18), scoring for these was limited to the FS-PDWI sequence for detailed assessments, while only overall scores were assigned to T1WI and T2WI.

#### 2.3.3 Lesion interpretation

Qualitative image analysis was conducted using PACS. Two radiologists (HL and MX) independently performed all imaging analyses. In cases of inconsistent interpretations, they resolved differences through consultation. There are six parts to the meniscus (19). The front horn, the body, and the posterior horn make up the medial and lateral menisci, respectively. A normal section receives one score, degeneration gets two, and a tear gets three on the evaluation scale. Additionally, there is a three-point scale (20) for evaluating ligaments, such as the patellar ligament, anterior and posterior cruciate ligaments, medial and lateral collateral ligaments, and each is rated as normal (1 point), sprained (2 points), or torn (3 points).

#### 2.3.4 Treatment evaluation

A sports medicine orthopedic doctor (HB, with 21 years of experience) conducted evaluations twice, with an interval exceeding 8 weeks, to determine the appropriate course of treatment—conservative or surgical—based on the overall imaging of meniscus and cruciate ligament tears.

#### 2.3.5 Statistical analysis

We used IBM’s SPSS 27.0 software, which is based in Armonk, NY, United States, to conduct our statistical studies. The mean and standard deviation are ways to display quantitative data that follows a normal distribution. Using a one-way analysis of variance (ANOVA) and the least significant difference (LSD) test, we checked for variance homogeneity. For data with different variances, Dunnett’s T3 tests were utilized. Scores for picture quality and outcomes of meniscus and ligament interpretations in the AIIA and SD groups were analyzed using the rank sum test. The intraclass correlation coeficient (ICC) test evaluated the consistency of these assessments within and between groups by two doctors (evaluated twice by the same doctor). Compared the internal and external consistency of these tests; results ranged from moderately consistent (0.41–0.60) to extremely consistent (0.61–0.80) to nearly fully consistent (0.81–1.00). Statistically significant differences were indicated by *p*-values <0.05.

## 3 Results

### 3.1 Scanning time

In the SD group: T1WI took 61 s, T2WI 60 s, FS-PDWI 115 s, totaling 466 s for the entire knee scan. In the AIIA group: T1WI took 27 s, T2WI 33 s, FS-PDWI 84 s, totaling 312 s for the complete knee joint scan.

### 3.2 Objective indicators results

In comparison to the SD group, the AIIA group had a considerably greater SNR of T1WI for the femur and subpatellar fat pad (*p* = 0.04 and *p* < 0.001, respectively), but the SD group had a significantly better SNR of T2WI for the femur (*p* = 0.004). Other noise, CNR, and SNR levels did not show any significant changes (*p* = 0.06–0.92) (Table 2).

**Table 2:** Noise and SNR of each group.

Group	T1WI					T2WI					FS-PDWI				
	F-noise	F-SNR	SFP-noise	SFP-SNR	CNR	F-noise	F-SNR	SFP-noise	SFP-SNR	CNR	F-noise	F-SNR	SFP-noise	SFP-SNR	CNR
SD	193.33 ± 45.40	31.04 ± 6.81	374.67 ± 129.22	18.94 ± 9.01	16.36 ± 8.68	206.71 ± 48.13	29.68 ± 6.98	428.95 ± 147.08	21.69 ± 8.18	20.57 ± 7.94	85.74 ± 18.05	16.67 ± 3.97	137.62 ± 55.25	18.99 ± 5.32	16.374 ± 4.74
AIIA	200.91 ± 48.68	33.98 ± 8.57	393.10 ± 130.78	24.60 ± 7.70	18.9 ± 6.43	192.72 ± 42.86	26.91 ± 7.14	357.03 ± 121.51	21.87 ± 11.26	20.01 ± 10.92	89.73 ± 16.18	15.44 ± 3.12	135.70 ± 59.12	17.39 ± 6.28	14.97 ± 6.19
F	0.79	4.39	0.61	13.91	3.54	2.88	4.69	8.67	0.10	0.10	1.66	3.65	0.03	2.30	1.97
P	0.38	0.04	0.44	0.001	0.06	0.09	0.03	0.004	0.92	0.75	0.20	0.06	0.85	0.13	0.16

SNR, signal-to-noise-ratio; SD, standard; AIIA, artificial intelligence-assisted iterative algorithm protocols; F-noise, femur noise, F-SNR, femur signal-to-noise ratio, SFP-noise, subpatellar fat pad noise, SFP-SNR, subpatellar fat pad signal-to-noise ratio.

### 3.3 Subjective indicators results

SD category. When it came to other types of noise and SNR levels, neither of the radiologists saw any noteworthy variations. For the SD group, the scores were: FS-PDWI overall score ICC = 0.65; FS-PDWI meniscus score ICC = 0.66; FS-PDWI ligamenta score ICC = 0.83; T1WI overall score ICC = 0.84; T2WI overall score ICC = 0.73. For the SD group, the scores were: FS-PDWI overall score ICC = 0.79; FS-PDWI meniscus score ICC = 0.83; FS-PDWI ligament score ICC = 0.78; T1WI overall score ICC = 0.90; T2WI overall score ICC = 0.87. Only the overall scores for two cases in T1WI and one case in FS-PDWI were 2 points, all others were ≥3 points, predominantly 4 and 5 points (Table 3). There was a significant difference between the AIIA and SD groups in terms of image quality scores for each sequence (*p* < 0.05, Table 3 and Figures 2, 3).

**Table 3:** Score of each group.

Dose group	FS-PDWI overall score	FS-PDWI meniscus score	FS-PDWI ligamenta score	T1WI overall score	T2WI overall score
SD	4.17 ± 0.05	4.20 ± 0.05	4.48 ± 0.05	3.90 ± 0.04	3.98 ± 0.06
AIIA	4.61 ± 0.05	4.61 ± 0.04	4.72 ± 0.04	4.04 ± 0.04	4.26 ± 0.05
Z	−5.88	−5.43	−3.58	−2.20	−3.61
Sig	0.001	0.001	0.001	0.03	0.001

SD (FS-PDWI overall score ICC = 0.65; FS-PDWI meniscus score ICC = 0.66; FS-PDWI ligamenta score ICC = 0.83; T1WI overall score ICC = 0.84; T2WI overall score ICC = 0.73); AIIA (FS-PDWI overall score ICC = 0.79; FS-PDWI meniscus score ICC = 0.83; FSPDWI ligamenta score ICC = 0.78; T1WI overall score ICC = 0.90; T2WI overall score ICC = 0.87).

### 3.4 Lesion interpretation results

Ligament: ICC = 0.74 in the SD group and 0.80 in the AIIA group. When it came to meniscus injuries, the SD group had an ICC of 0.87 and the AIIA group had an ICC of 0.82, but there was no statistically significant difference in how the two groups interpreted ligament injuries (*z* = −0.47, *p* = 0.64). The groups’ interpretations of meniscus injuries were not significantly different from one another (*z* = −1.46, *p* = 0.15).

### 3.5 Treatment evaluation

The evaluations of treatment outcomes, based on imaging from both the SD and AIIA groups, were highly consistent. The evaluations included 11 surgical treatments and 50 conservative treatments in both groups, showing high consistency.

## 4 Discussion

The total duration of MRI knee scans was reduced from 466 s to 312 s. Subjective evaluations of the reconstructed images showed that the AIIA group had better quality, even though the two groups did not differ in terms of noise, SNRs, and CNRs (Figures 3, 4). Figures 3C-2, 4C-2 show the results of these improvements to the picture quality, contrast, and sharpness, as well as the reduction of motion artifacts in MRI scans of the knee. Additionally, AIIA facilitated the production of high-resolution, nearly isotropic images of the knee MRI, ensuring optimal quality. There was no difference in the diagnosis of meniscus and ligament injuries or in formulating treatment plans for the knee joint between the SD and AIIA groups. This technology also enabled the generation of high-quality reformatted images from the same acquisition phase.

The total efficiency and diagnostic capabilities of MRI scans are greatly affected by the optimization of acceleration level, image quality, and scan time (21). While using too much acceleration could cause scans to take too long, using too little can ruin images and lead to wrong diagnoses. Careful evaluation of the ideal acceleration level within the range of scanning parameters is required (19). Due to its high in-plane spatial resolution and strong tissue contrast, FS-PDWI and T1WI are the gold standards for knee MRI (22, 23). A multi-plane 2D-TSE sequence is now the gold standard for clinical MRI knee exams. This process usually takes around 15 min (24) because of all the different planes and contrasts. In this study, the T1WI AIIA sequence took 27 s, the T2WI scan 33 s, the FS-PDWI sequence 84 s, totaling approximately 5 min for the entire knee joint scan—merely one-third of the time required for conventional scanning.

Compared with other knee joint accelerated scanning techniques, three teams used 3.0 T MRI scanners in their studies. The team of Iuga et al. (24) analyzed the FS-PDWI sequence with a scanning time of approximately 114 s and an image quality rating of 4.55 using a 5-point scale. Our team achieved a scanning time of 84 s with an image quality rating of 4.6. The team of Kim et al. (25) employed AI technology for scanning, their T2WI sequence of the knee joint took 51 s, compared to 33 s achieved by our team. Both images were judged as excellent. The team of Herrmann et al. (23) also utilized AI technology, recording scanning times for the T1WI sequence at 91 s and the FS-PDWI sequence at 93 s, both longer than those of our team. The knee joint images from both teams met clinical requirements.

Our team uniquely assessed all three sequences required for comprehensive knee joint scanning using AIIA technology, unlike other teams who studied only one or two sequences (16, 22–25). This approach makes our research more comprehensive. Historically, accelerated knee joint scanning relied on a single AI technology (22–25). Recently, there has been a trend towards combined research on accelerated scanning using two technologies (16). This study employs a combination of multiple technologies to accelerate MRI scanning, starting with optimizing K-space, followed by iterative image reconstruction, and enhancing image quality using AI technology, thus significantly reducing MRI scanning time.

It is important to highlight a few caveats of this research. To begin, just one vendor’s 3.0-T MRI scanner was used for image acquisition, and no other devices were validated. To thoroughly evaluate the scalability of accelerated technology with AI assistance, additional research with other vendors and scanning locations is required. Secondly, we did not compare the diagnostic accuracy of the SD and AIIA pictures to a reference knee arthroscope, but we did evaluate their quality. Lastly, there is a possibility that the measured values are skewed due to selection bias due to the limited sample size.

This study demonstrates the feasibility of using the AIIA protocol for knee imaging, which effectively reduces MRI scanning time while maintaining image quality. Consequently, this method is a practical option for clinical application.

## Data Availability

The original contributions presented in the study are included in the article/supplementary material, further inquiries can be directed to the corresponding authors.
